# Selection based on the size of the black tie of the great tit may be reversed in urban habitats

**DOI:** 10.1002/ece3.999

**Published:** 2014-06-07

**Authors:** Juan Carlos Senar, Michael J Conroy, Javier Quesada, Fernando Mateos-Gonzalez

**Affiliations:** 1 Natural History Museum of BarcelonaBarcelona, Spain; 2 Warnell School of Forestry and Natural Resources, University of GeorgiaAthens, Georgia; 3 Department of Ecology and Evolution, Evolutionary Biology Centre, University of UppsalaUppsala, Sweden

**Keywords:** Divergent selection, *Parus major*, plumage coloration, survival, trap response, urban adaptation

## Abstract

A standard approach to model how selection shapes phenotypic traits is the analysis of capture–recapture data relating trait variation to survival. Divergent selection, however, has never been analyzed by the capture–recapture approach. Most reported examples of differences between urban and nonurban animals reflect behavioral plasticity rather than divergent selection. The aim of this paper was to use a capture–recapture approach to test the hypothesis that divergent selection can also drive local adaptation in urban habitats. We focused on the size of the black breast stripe (i.e., tie width) of the great tit (*Parus major*), a sexual ornament used in mate choice. Urban great tits display smaller tie sizes than forest birds. Because tie size is mostly genetically determined, it could potentially respond to selection. We analyzed capture/recapture data of male great tits in Barcelona city (*N* = 171) and in a nearby (7 km) forest (*N* = 324) from 1992 to 2008 using MARK. When modelling recapture rate, we found it to be strongly influenced by tie width, so that both for urban and forest habitats, birds with smaller ties were more trap-shy and more cautious than their larger tied counterparts. When modelling survival, we found that survival prospects in forest great tits increased the larger their tie width (i.e., directional positive selection), but the reverse was found for urban birds, with individuals displaying smaller ties showing higher survival (i.e., directional negative selection). As melanin-based tie size seems to be related to personality, and both are heritable, results may be explained by cautious personalities being favored in urban environments. More importantly, our results show that divergent selection can be an important mechanism in local adaptation to urban habitats and that capture–recapture is a powerful tool to test it.

## Introduction

Understanding and modeling how selection shapes phenotypic traits is central to evolutionary ecology. One of the main approaches to model selection is to relate the appearance of the trait to individual survival, taking the trait as an individual covariate and survival as an overall measurement of individual fitness (Gimenez et al. 2008; Cam 2009; Conroy 2009).

Since individuals in natural populations cannot be followed exhaustively, it is widely recognized that detectability has to be incorporated into models, and capture–recapture of tagged individuals has become the most widely used standard tool for that (Williams et al. 2002). The event that a marked individual is observed (generally, captured but sometimes resighted) reflects both the probability of the individual surviving to the time of sampling and the probability of recapture conditional on survival. If both of these probabilities are related to the same phenotypic trait, it can be difficult to distinguish effects on survival probabilities from effects on recapture probabilities, unless the trait is modelled as a covariate in both probabilities (Kingsolver and Smith 1995; Zabel et al. 2005).

Great tits *Parus major* display a melanin-based black breast stripe that we call a tie. Birds with larger ties are more dominant and aggressive (Järvi and Bakken 1984; Pöysä 1988). Males with large ties have also been found to defend their nests more intensely against predators (Norris 1990b; Quesada and Senar 2007) and to show high levels of nest attentiveness (Norris 1990b). Additionally, male great tits with large ties have been found to pair with females that produce eggs with a high yolk mass, lay large clutches, and have offspring that show greater viability (Norris 1990a, 1993; Remes 2011). The black tie is therefore thought to be a signal of individual quality and to be used by females in mate choice (Norris 1990a,b, 1993; Quesada and Senar 2009). As a consequence, we should expect tie size to covary with survival. However, as tie size is also related to dominance (Järvi and Bakken 1984; Pöysä 1988), which in turn has been shown to strongly affect recapture rates (Summerlin and Wolfe 1973; Drickamer et al. 1999), we should also expect tie size to covary with recapture rate. Tie size can therefore provide a nice example of a study of selection where recapture probabilities should be modelled in a manner analogous to the survival function (Kingsolver and Smith 1995).

Of the different forms of selection, divergent selection is one of the most interesting, because it is a powerful mechanism in local adaptation and ecological speciation (Futuyma 1998; Nosil 2012). One of the main sources of divergent selection stems from differences between environments (Schluter 2000; Rundle and Nosil 2005). In spite of its interest, however, this form of selection has never been analyzed using a capture–recapture approach, where divergent selection should be tested by a significant interaction in survival estimates between habitat and the trait in question.

A particularly interesting process in ecological speciation refers to traits under divergent selection that also contribute to nonrandom mating. Such traits have been called “magic traits”, because of the fact that a single trait performs the functions normally attributed to two separate traits (Gavrilets 2004; Servedio et al. 2011). Typically, these traits are caused by divergent selection acting on mating cues, such as color or body size (Servedio et al. 2011).

Urban habitats have grown rapidly in recent decades (Marzluff et al. 2001; Gaston 2010). In spite of the traditional belief that urban habitats are less suitable for wildlife, there is increasing evidence that species may adapt to urban environments and that these may be considered as new habitats, available for colonization and local adaptation (Shochat et al. 2006; Evans 2010). Recent research on urban ecology suggests that local variation in environmental conditions between urban and natural areas can prompt marked trait divergence between close populations (Evans 2010). Consequently, urban populations may differ from natural populations in many traits such as demography, morphology, communication, physiology, and genetic structure (Evans 2010). The comparison between urban and natural habitats may therefore be particularly fruitful to detect patterns of divergent selection and hence ecological speciation. Until now, however, most examples of differences between urban and nonurban animals refer to processes of behavioral plasticity rather than divergent selection (Sol et al. 2013).

Great tits *Parus major* inhabiting urban habitats have been shown to differ genetically and morphologically from their forest or rural counterparts (Schmidt 1986; Björklund et al. 2010). Gene flow between nearby urban and forest habitats is also very low (Björklund et al. 2010). This species and its use of urban habitat may therefore be ideal to test for divergent selection. Preliminary analyses have shown that forest great tits display larger tie sizes than urban great tits. As tie size is mostly genetically determined (Norris 1993; Quesada and Senar 2009), this trait is potentially subject to selection. Because tie size additionally seems to contribute to nonrandom mating (Norris 1990a,b, 1993; Quesada and Senar 2009; Remes and Matysiokova 2013), this trait could conform to a “magic trait”.

The aim of this paper was to analyze individual variation and patterns of selection on tie size in forest and urban habitats, and to determine, using capture–recapture methodology, the relationship between tie size, habitat, and survival rates. As tie size can also affect recapture probabilities, we modelled both survival and recapture probabilities as a function of tie width (Kingsolver and Smith 1995; Zabel et al. 2005). We predicted that selection, as measured by differential survival in relation to tie width, would differ between forest and urban birds, explaining why forest birds display larger ties than urban birds.

## Materials and Methods

Urban great tits were trapped within Desert de Sarria area, in Barcelona city, which is close to the park of Setmenat (Björklund et al. 2010). The area has a suburban structure, with some large buildings, houses, and gardens. We trapped a total of 171 male great tits in this area between 1992 and 2005. The forest sampling area was located in the Can Cata field station, a Mediterranean mixed forest dominated by deciduous (*Quercus cerrioides*) and evergreen oak (*Quercus ilex*) at the bottom of the valleys and Aleppo pine (*Pinus halepensis*) forest on the hills. This field station is located seven kilometer from the urban sampling area. We trapped a total of 324 male great tits in the forest from 1998 to 2008.

Birds were captured using funnel baited traps (Senar et al. 1997). The sample we used included birds trapped from January to June. Sex and age of birds were determined according to Svensson (1992) and Jenni and Winkler (1994). We distinguished two age classes: young birds (also called yearlings) (Euring codes 3 and 5, after their partial molt and before their first complete postbreeding molt) and adults, defined as birds known to have hatched at least 2 years before the calendar year of capturing (Euring codes >5). The size of the black tie was measured as the width of the tie at the level of the clavicle, following Järvi and Bakken (1984) and Pöysä (1988). It is known that repeatability and accuracy when measuring great tit tie size are higher when digital photographs of the whole tie are taken and analyzed using a program that allows measurement of the size of colored patches (Figuerola and Senar 2000; Quesada and Senar 2007). However, photographs were not available for the earliest years of the study. To increase repeatability (Harper 1994), we used the average of the measures of tie width obtained for each individual throughout the study (mean 3.1 measures per individual, SE = 0.27, range 1–33 measures per individual). A subsample of birds measured in 2012–2013 showed tie width, measured at the level of the clavicle, to be highly correlated to tie size measured from digital photographs (*r* = 0.84, *P* < 0.001, *N* = 129).

Presence of divergent selection on tie width in the two habitats (forest vs. city) was assessed using survival as an overall measure of fitness in relation to tie width, as an individual covariate. Survival analysis was based on Cormack–Jolly–Seber models (CJS). We used the R-MARK program (White and Burnham 1999) to model local survival rates and to analyze the influence of individual covariates and their interactions on local survival. Analyses were restricted to males because these individuals display larger and greater variation in tie width. As the sampling period differed for the urban (1992–2005) and the forest area (1998–2008), we analyzed each data set separately. The analysis procedure was, however, the same for each of the two data sets. Model selection started from a global model where both survival and recapture probability varied according to time. The fit of the global CJS model to the data was assessed by the Release program goodness of fit (Burnham et al. 1987). The overdispersion parameter (c-hat) was calculated using the bootstrapping approach available in MARK. Overdispersion was mild (c-hat forest = 1.02; city = 1.12) and thus there was no indication of violation of the assumption that fates of the individuals were independent of each other (Anderson et al. 1994). We included age in the global model to test for age effects. Models incorporating age-dependent effects allowed survival parameters estimated from birds captured as young to be different from adults only during the year after first capture. Because young birds become adults in the 2nd year, and by definition there are no recaptures of young birds, recapture rate was not modelled as a function of age. Analyses showed age to have some effect in urban birds, but not in forest birds (see Results).

Model simplification started by analyzing the factors affecting recapture probability by constructing models with and without time-specific variation. Because tie width has been found to be related to dominance (Järvi and Bakken 1984; Pöysä 1988) and this may affect trapping probability (Summerlin and Wolfe 1973; Drickamer et al. 1999), we additionally modelled recapture probability as a function of tie width. In a second step of model simplification, we used the top model(s) for recapture rate and analyzed variation in survival. Finally, using the capture and survival simplified model, we tested the possible relationship between survival and individual tie width.

We used Akaike's information criterion corrected for overdispersion (c-hat) and sample size (QAICc) for model selection (Anderson et al. 1994; Burnham and Anderson 2002). Models with lowest QAICc are assumed to best fit the data with the least possible number of parameters. Models with QAICc values differing by <2 were considered equivalent.

To test for a difference in slopes in survival rates in relation to tie width in the two habitats, we jointly modelled the two data sets, formatting data with two groups (forest and urban). We formatted that no birds had been trapped in the forest habitat from 1992 to 1997 by typing 0 in the capture history of the forest habitat for that period, and in 2006–2008, for the urban habitat. We then compared a model including a group + tie width effect for survival, to the model also including the interaction: group + tie width + group * tie width.

## Results

**Figure 1:**
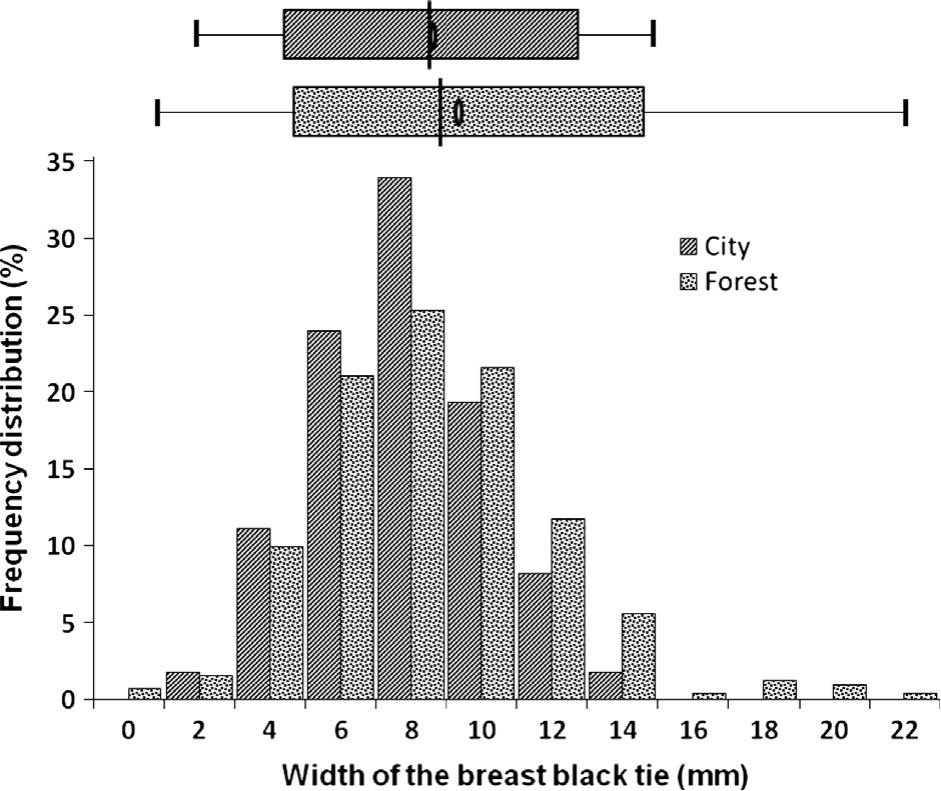
Frequency distribution (%) and box-and-whisker plot showing the mean (ellipses), the median (lines), 5th and 95th percentiles and nonoutlier range of the size of the black breast tie of forest and urban male great tits. Mean (±SE) values of tie size for urban birds: 8.6 ± 0.23 mm and for forest birds: 9.3 ± 0.17 mm. The size of the black tie was measured as the width of the tie at the level of the clavicle (1992–2008).

The width of the black tie of forest great tits was larger than that of urban birds (*F*_1,493_ = 6.16, *P* = 0.01) (Fig. 1).

**Figure 2:**
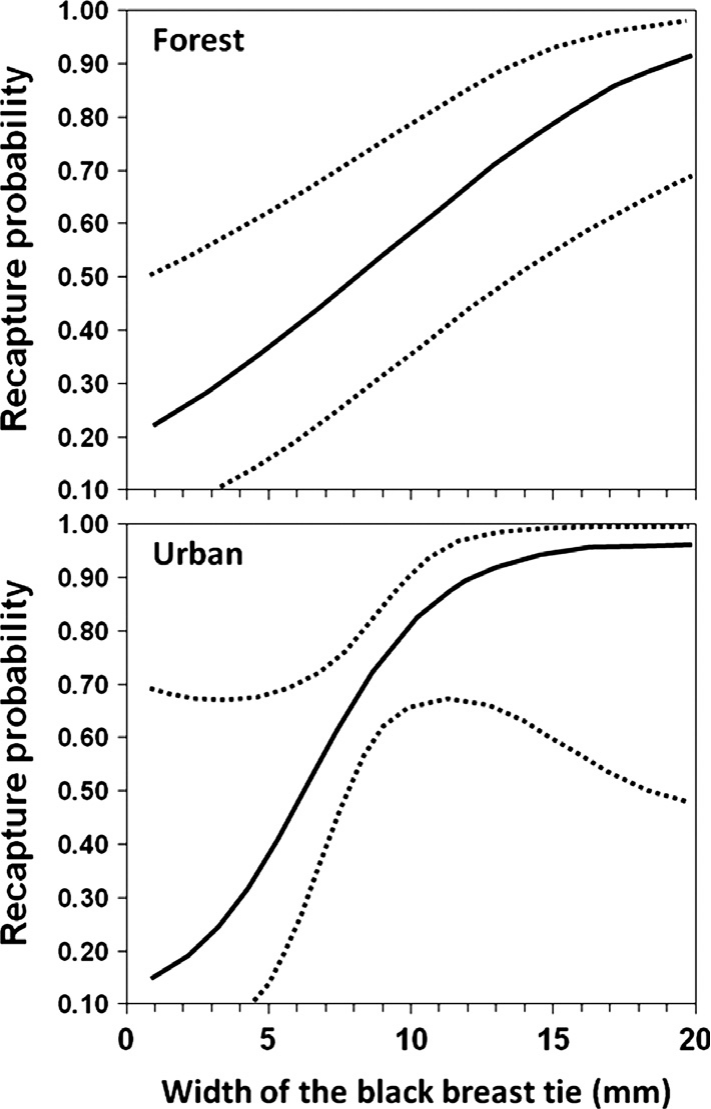
Variation in recapture probability of male great tits in relation to the size of their black breast tie. Data from forest and urban birds are provided separately.

Capture–recapture data showed a good fit to a general CJS model, for both data sets (Table 1). There was no evidence of an age effect on survival in the forest habitat (Table 2). However, some age effect was apparent for the urban habitat (Table 3). When modelling recapture probability for the forest data, the lowest QAICc was for the model including time variation but also an additive effect of tie width (Table 2). In the urban habitat, there was also an effect of tie width on recapture rate, although not so strong as in the forest habitat (Table 3). Recapture probability could therefore be modelled in both habitats as increasing with tie width (Fig. 2).

**Table 1:** Results of the Release test about the adjustment of the CJS model to the forest and urban data sets. Data showed a good fit to a general CJS model

Test	Chi-square	df	*P*
Forest			
Test 2	6.92	8	0.55
Test 3.SR	6.03	9	0.74
Test 3.Sm	8.67	7	0.28
Test 3	14.7	16	0.55
Test 2 + 3	21.62	24	0.60
Urban			
Test 2	2.15	9	0.99
Test 3.SR	5.75	12	0.93
Test 3.Sm	5.91	9	0.75
Test 3	11.66	21	0.95
Test 2 + 3	13.82	30	0.99

**Table 2:** Model selection for time, age, and tie width effects on recapture and survival probability in forest great tit males. Models are ranked according to QAICc values

	npar	QAICc	DeltaQAICc	Weight	QDeviance
Modelling time and age effects/forest					
Phi(*t*) p(*t*)	20	821.10	0.0	0.35	156.17
Phi(*t*) p(.)	11	822.27	1.2	0.20	176.57
Phi(.) p(*t*)	11	822.82	1.7	0.15	177.12
Phi(age + *t*) p(*t*)	21	823.15	2.0	0.13	156.03
Phi(age + *t*) p(.)	12	823.67	2.6	0.10	175.87
Phi(age) p(*t*)	12	824.88	3.8	0.05	177.08
Phi(age * *t*) p(.)	21	826.54	5.4	0.02	159.42
Phi(age * *t*) p(*t*)	30	830.59	9.5	0.00	143.41
Phi(.) p(.)	2	856.60	35.5	0.00	229.42
Phi(age) p(.)	3	858.56	37.5	0.00	229.36
Modelling recapture					
Phi(.) p(*t* + tie)	12	787.99	0.0	0.80	763.34
Phi(*t*) p(*t* + tie)	21	791.21	3.2	0.16	747.25
Phi(.) p(*t* * tie)	21	793.91	5.9	0.04	749.95
Phi(*t*) p(*t* * tie)	30	798.62	10.6	0.00	734.59
Phi(*t*) p(tie)	12	801.09	13.1	0.00	776.44
Phi(*t*) p(*t*)	20	813.46	25.5	0.00	154.64
Phi(*t*) p(.)	11	814.43	26.4	0.00	174.84
Phi(.) p(*t*)	11	814.98	27.0	0.00	175.38
Phi(.) p(tie)	3	831.29	43.3	0.00	825.24
Phi(.) p(.)	2	848.24	60.3	0.00	227.18
Modelling survival					
Phi(tie) p(*t* + tie)	13	782.93	0.0	0.85	756.17
Phi(.) p(*t* + tie)	12	787.99	5.1	0.07	763.34
Phi(*t* + tie) p(*t* + tie)	22	788.27	5.3	0.06	742.12
Phi(*t*) p(*t* + tie)	21	791.21	8.3	0.01	747.25
Phi(*t* * tie) p(*t* + tie)	31	793.08	10.1	0.01	726.77

Phi, survival probability; p, recapture probability; time (*t*), parameters are allowed to change between capture occasions; age, parameters are allowed to change according to age of the birds (young and adult birds); tie, width of the black breast band; +, only main factors included in the model; *, main factors and its interaction included in the model.

**Table 3:** Model selection for time and tie width effects on recapture and survival probability in urban great tit males. Models are ranked according to QAICc values

	npar	QAICc	DeltaQAICc	Weight	QDeviance
Modelling time and age effects/urban					
Phi(.) p(.)	2	464.74	0.0	0.61	183.43
Phi(age) p(.)	3	465.75	1.0	0.37	182.39
Phi(.) p(*t*)	14	472.02	7.3	0.02	165.15
Phi(age) p(*t*)	15	473.06	8.3	0.01	163.96
Phi(*t*) p(.)	14	476.01	11.3	0.00	169.14
Phi(age + *t*) p(.)	15	478.24	13.5	0.00	169.14
Phi(*t*) p(*t*)	26	487.32	22.6	0.00	152.46
Phi(age + *t*) p(*t*)	27	489.34	24.6	0.00	152.02
Phi(age * *t*) p(.)	27	494.07	29.3	0.00	156.75
Phi(age * *t*) p(*t*)	39	508.11	43.4	0.00	139.73
Modelling recapture					
Phi(.) p(tie)	3	464.15	0.0	0.36	458.06
Phi(.) p(.)	2	464.74	0.6	0.27	183.43
Phi(age) p(tie)	4	465.25	1.1	0.21	457.10
Phi(age) p(.)	3	465.75	1.6	0.16	182.39
Modelling survival					
Phi(age + tie) p(tie)	5	460.64	0.0	0.30	450.42
Phi(tie) p(tie)	4	460.93	0.3	0.26	452.79
Phi(age * tie) p(tie)	6	461.06	0.4	0.24	448.75
Phi(.) p(tie)	3	464.15	3.5	0.05	458.06
Phi(.) p(.)	2	464.74	4.1	0.04	183.43
Phi(age) p(tie)	4	465.25	4.6	0.03	457.10
Phi(age) p(.)	3	465.75	5.1	0.02	182.39
Phi(tie) p(.)	3	465.76	5.1	0.02	459.67
Phi(age + tie) p(.)	4	466.70	6.1	0.01	458.56
Phi(age * tie) p(.)	5	467.61	7.0	0.01	457.39

Phi, survival probability; p, recapture probability; time (*t*), parameters are allowed to change between capture occasions; age, parameters are allowed to change according to age of the birds (young and adult birds); tie, width of the black breast band; +, only main factors included in the model; *, main factors and its interaction included in the model.

**Figure 3:**
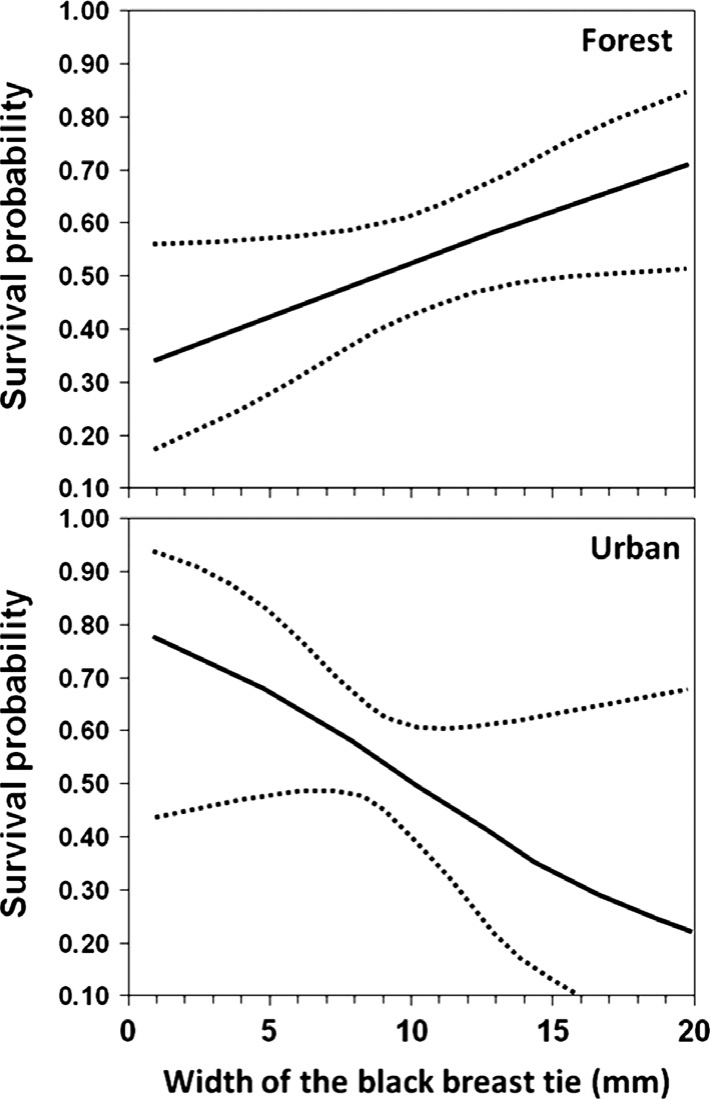
Variation in survival rate of male great tits in relation to the size of their black breast tie. Data from forest and urban birds are provided separately. While survival increased with tie size in the forest, the relationship was reversed in the urban habitat.

When modelling survival rate, we found it to be dependent on tie width in both habitats (Tables 2 and 3). However, while survival increased with tie width in the forest, in the urban habitat, the relationship was reversed, so that survival decreased with tie width (Fig. 3). We tested for a difference in slopes by using AIC to compare, in a full model including both datasets, the model including the interaction between habitat and tie width in survival rate and the model with no interaction. As the model including the interaction had an AIC value that was smaller than that of the model with no interaction, we concluded that the slopes between the two habitats differed (Table 4, Fig. 3). This was true in models with and without recapture probabilities modelled as a function of tie width.

**Table 4:** Model selection for time and tie width effects on recapture and survival probability in forest and urban great tit males, specifically testing for a difference in slopes in survival rate in the two habitats. Models are ranked according to AICc values

Model	AICc	Delta AICc	AICc Weights	Model Likelihood	Num. Par	Deviance
Phi(g + *t* + tie + group * tie) p(g * *t* + tie)	1314.10	0.0	0.98	1	40	1229.76
Phi(g + *t* + tie) p(g * *t* + tie)	1321.70	7.6	0.02	0.023	39	1239.57
Phi(g + *t* + tie + group * tie) p(g * *t*)	1328.14	14.0	0.00	0.001	38	1248.23
Phi(g + *t* + tie) p(g * *t*)	1337.08	23.0	0.00	0	37	1259.38

Phi, survival probability; p, recapture probability; time (*t*), parameters are allowed to change between capture occasions; tie, width of the black breast band; g, group, referring to forest and urban birds; +, only main factors included in the model; *, main factors and its interaction included in the model.

A better fit of the interaction (*) model than the additive model (+) indicates a difference in slopes.

## Discussion

The width of the black breast tie of Barcelona urban great tits was smaller than that of forest birds. As eumelanin-based traits are costly to produce (Galvan and Alonso-Alvarez 2008) and may be environmentally constrained (Fargallo et al. 2007; Galvan et al. 2010), the smaller width of the melanin-based breast tie of urban great tits could be a by-product of the higher contamination and reduced food availability in these habitats (Gaston 2010). However, this is not necessarily the case, as for example in other nonurban highly contaminated areas, great tits displayed larger ties than those in noncontaminated habitats (Dauwe and Eens 2008).

An alternative possibility is that as tie width is to some degree heritable (Norris 1993; Quesada and Senar 2009), and gene flow between forest and urban populations is very small (Björklund et al. 2010), tie width could be subjected to divergent selection in these two habitats. Differences between environments are one of the main sources of divergent selection (Schluter 2000; Rundle and Nosil 2005). Analysis of tie width in relation to survival rate strongly suggested that this was the case. Tie width was positively selected in the forest habitat, with individuals of larger tie width enjoying a higher survival, while in the urban habitat, the relationship was reversed, and tie width was negatively selected. This conclusion is supported by the significant interaction between habitat and the trait in question.

Urban habitats differ greatly from natural areas, and they have been shown to specially exert selection pressures which could facilitate the presence of divergent selection when compared with natural habitats (Shochat et al. 2006; Evans 2010; Sol et al. 2013). However, the challenge is to determine the mechanism by which, in our case, small ties are favored in the city while the reverse is the case in forests.

At this stage, it is difficult to ascertain this mechanism. One possibility could be that tie width reflected personality, defined as consistent individual differences in behavior related to exploration, caution, and neophobia (Andrew et al. 2004; Dall et al. 2004; Reale et al. 2007), and that this was the true target of selection, indirectly favoring different tie widths in different environments. The fact that tie width was strongly positively related to recapture rate, a behavioral response reflecting personality (Boon et al. 2008; Biro and Dingemanse 2009; Garamszegi et al. 2009; Carter et al. 2012), supports this view. More data are of course needed to demonstrate a link between tie width and personality in great tits. Additionally, the mechanism favoring different tie widths in different environments could lie in other behavioral or physiological traits in addition to personality.

The link between melanin-based coloration and several physiological, morphological, and behavioral traits (Roulin 2004) has been proposed to originate in the pleiotropic effects of the genes regulating the synthesis of melanin (Ducrest et al. 2008). Pleiotropy has been recognized as an important genetic mechanism favoring local adaptation and ecological speciation (Nosil 2012). Hence, although the black tie is probably not the direct subject of selection, it could be in fact under true divergent selection via pleiotropic effects. As tie size seems to contribute to nonrandom mating (Norris 1990a,b, 1993; Quesada and Senar 2009; Remes and Matysiokova 2013), it could be considered a “magic trait” (Servedio et al. 2011). However, the question now appears of whether urban great tit females have realized that in the urban habitat, the “high quality” males are those with small ties, and preferentially select for these males during mate choice. The question then is whether urban great tit females have entered into an evolutionary trap (Schlaepfer et al. 2002) or whether, on the contrary, a plastic mate choice strategy has evolved (Qvarnström et al. 2000).

Modeling how selection shapes phenotypic traits is central to evolutionary biology, and the capture–recapture methodology has provided in recent years a powerful approach (Gimenez et al. 2008; Cam 2009; Conroy 2009). The study of divergent selection, however, is more complex than just relating the appearance of a trait (individual covariate) to individual survival (Gimenez et al. 2008; Cam 2009; Conroy 2009). We have to test for a significant interaction in survival estimates between habitat and the trait in question. If the interaction is significant, as in this paper, results are very robust. The capture–recapture approach also allows modelling both survival and recapture rates as a function of the trait under study. When we analyze traits which are highly related to behavior and hence can affect both survival and trapping probability, approaches not using the capture–recapture technique can be highly biased (Kingsolver and Smith 1995; Zabel et al. 2005). This was the case of tie width, which was the target of selection but also highly affected trapability in both localities. Hence, whatever the answer may be to why different tie widths may be favored in different environments, our work stresses the utility of the capture–recapture approach to test hypotheses about divergent selection.
